# Cystadénome mucineux pancréatique doublement compliqué de pancréatite aigüe et de rupture dans le rétro-péritoine

**DOI:** 10.11604/pamj.2017.28.203.13715

**Published:** 2017-11-06

**Authors:** Houcine Maghrebi, Amine Makni

**Affiliations:** 1 Département de Chirurgie, Hôpital La Rabta, Faculté de Médecine de Tunis, Tunis, Tunisie

**Keywords:** Cystadénome mucineux, pancréatite aigüe, chirurgie, imagerie, Mucinous cystadenoma, acute pancreatitis, surgery, imaging

## Abstract

Mucinous cystadenomas are benign tumors with malignant potential. They are often revealed by non-specific abdominal pain, jaundice or an episode of acute pancreatitis. We here report an exceptional case of mucinous cystadenoma doubly complicated by acute pancreatitis and retroperitoneal rupture. The study involved a 30-year old non-weighted female patient, presenting with epigastric pain associated with left hypochondrium evolving over the last three months and which had intensified without fever or jaundice in the last 3 days. Clinical examination showed impingement on palpation of the epigastrium and of the left hypochondrium. There was no palpable mass. Laboratory tests were without abnormalities, except for lipasemia that was 8-times the upper normal. Abdominal CT scan showed bi-loculated cystic mass in the pancreas tail, measuring 111 mm * 73 mm, with a thin wall and a fluid content, associated with an infiltration of the left perirenal fascia. MRI (Panel A) showed mucinous cystadenoma with retroperitoneal rupture. The caudal portion of the main pancreatic duct was slightly dilated and communicated with the pancreatic cyst. The patient underwent surgery via bi-sub-costal approach. A cystic mass in the pancreas tail with retroperitoneal rupture associated with acute pancreatitis (outflow of necrotic content from left anterior prerenal space) was found. Caudal splenopancreatectomy was performed (Panel B). The postoperative course was uneventful. The anatomo-pathological examination of the surgical specimen showed pancreatic mucinous cystadenoma with low-grade dysplasia.

## Image en médecine

Les cystadénomes mucineux sont des tumeurs bénignes à potentiel malin. Ils sont souvent révélés par des douleurs abdominales non spécifiques, un ictère, ou un épisode de pancréatite aiguë. Nous rapportons l’observation exceptionnelle d’un cystadénome mucineux doublement compliqué de pancréatite aigue et de rupture dans le rétro-péritoine. Il s’agit d’une patiente âgée de 30 ans, non tarée, qui nous a consulté pour des douleurs épigastriques et de l’hypochondre gauche évoluant depuis trois mois et qui se sont accentuées depuis 3 jours, sans fièvre ni ictère. L’examen clinique avait noté un empâtement à la palpation de l’épigastre et de l’hypochondre gauche. Il n’y avait pas de masse palpable. Le bilan biologique était sans anomalie en dehors d'une lipasémie à 8 fois la normale. Une TDM abdominale a été pratiquée montrant une masse kystique de la queue du pancréas, bi-loculée de 111 mm x 73 mm, à paroi fine et à contenu liquidien, associé à une infiltration du fascia para rénal gauche. Un complément d’IRM (A) a été réalisé montrant un aspect de cystadénome mucineux rompu dans rétro-péritoine. La portion caudale du canal pancréatique principal était légèrement dilatée et communiquait avec le kyste pancréatique. La malade a été opérée par voie bi sous costale. Il s’agissait d’une formation kystique de la queue du pancréas rompue dans le rétro-péritoine associée à une pancréatite aigue (coulée de nécrose de l’espace pré rénal antérieur gauche). Il a alors été réalisé une splénopancréatectomie caudale (B). Les suites opératoires ont été simples. L’examen anatomo-pathologique de la pièce opératoire avait conclu à un cystadénome mucineux pancréatique siège de lésions de dysplasie de bas grade.

**Figure 1:**
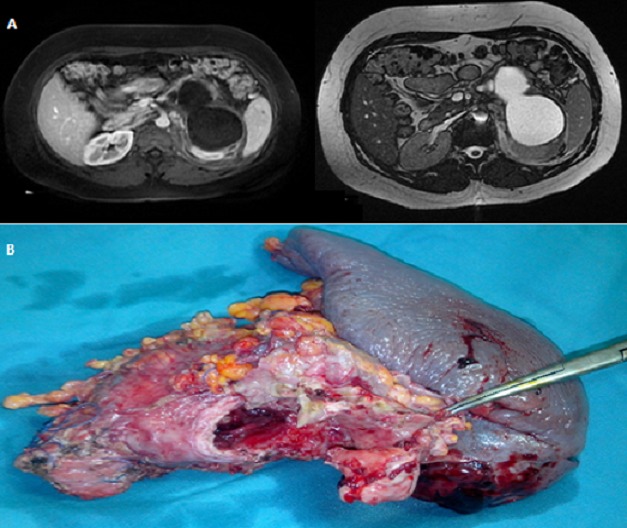
A) imagerie par résonance magnétique montrant le cystadénome mucineux rompu dans rétro-péritoine; B) pièce de spléno-pancréatectomie caudale

